# Insights in to the pathogenesis of axial spondyloarthropathy based on gene expression profiles

**DOI:** 10.1186/ar2855

**Published:** 2009-11-09

**Authors:** Srilakshmi M Sharma, Dongseok Choi, Stephen R Planck, Christina A Harrington, Carrie R Austin, Jinnell A Lewis, Tessa N Diebel, Tammy M Martin, Justine R Smith, James T Rosenbaum

**Affiliations:** 1 Casey Eye Institute, Oregon Health & Science University, 3181 SW Sam Jackson Park Road, Portland, Oregon, 97239, USA; 2 Department of Public Health & Preventive Medicine, Oregon Health & Science University, 3181 SW Sam Jackson Park Road, Portland, Oregon, 97239, USA; 3 Department of Cell & Developmental Biology, Oregon Health & Science University, 3181 SW Sam Jackson Park Road, Portland, Oregon, 97239, USA; 4 Department of Medicine, Oregon Health & Science University, 3181 SW Sam Jackson Park Road, Portland, Oregon, 97239, USA; 5 Gene Microarray Shared Resource, Oregon Health & Science University, 3181 SW Sam Jackson Park Road, Portland, Oregon, 97239, USA; 6 Department of Molecular Microbiology & Immunology, Oregon Health & Science University, 3181 SW Sam Jackson Park Road, Portland, Oregon, 97239, USA

## Abstract

**Introduction:**

Axial spondyloarthropathy (SpA) is a group of inflammatory diseases, with ankylosing spondylitis as the prototype. SpA affects the axial skeleton, entheses, joints and, at times, the eyes. This study tested the hypothesis that SpA is characterized by a distinct pattern of gene expression in peripheral blood of affected individuals compared with healthy controls.

**Methods:**

High-density, human GeneChip^® ^probe arrays were used to profile mRNA of peripheral blood cells from 18 subjects with SpA and 25 normal individuals. Samples were processed as two separate sets at different times (11 SpA + 12 control subjects in primary set (Set 1); 7 SpA+ 13 control subjects in the validation set (Set 2)). Blood samples were taken at a time when patients were not receiving systemic immunomodulatory therapy. Differential expression was defined as a 1.5-fold change with a q value < 5%. Gene ontology and pathway information were also studied.

**Results:**

Signals from 134 probe sets (representing 95 known and 12 unknown gene transcripts) were consistently different from controls in both Sets 1 and 2. Included among these were transcripts for a group of 20 genes, such as interleukin-1 (IL-1) receptors 1 and 2, Nod-like receptor family, pyrin domain containing 2 (NLRP2), secretory leukocyte peptidase inhibitor (SLPI), secreted protein acidic and rich in cysteine (SPARC), and triggering receptor expressed on myeloid cells 1 (TREM-1) that are clearly related to the immune or inflammatory response and a group of 4 transcripts that have a strong role in bone remodeling.

**Conclusions:**

Our observations are the first to implicate SPARC, SLPI, and NLRP2, a component of the innate immune system, in the pathogenesis of SpA. Our results also indicate a possible role for IL-1 and its receptors in SpA. In accord with the bone pathology component of SpA, we also found that expression levels of transcripts reflecting bone remodeling factors are also distinguishable in peripheral blood from patients with SpA versus controls. These results confirm some previously identified biomarkers implicated in the pathogenesis of SpA and also point to novel mediators in this disease.

## Introduction

Axial spondyloarthropathy (SpA) is a family of polygenic inflammatory diseases for which the pathophysiology is complex, with much remaining unknown. Ankylosing spondylitis is the most common form of SpA. Study of gene expression using microarrays offers a novel approach to determining pathogenesis of diseases. Analysis of peripheral blood in patients with systemic lupus erythematosus using this technique has led to the discovery that many lupus patients have an upregulation of genes induced by type I interferons [1].

The present study utilizes a methodology that incorporates an experimental design consisting of primary and validation datasets of subjects, a comprehensive microarray platform, and robust statistical techniques to investigate the presence of a SpA gene expression signature and the presence of novel biomarkers of disease.

## Materials and methods

### Subjects

This study is in compliance with the Helsinki Declaration and was approved by the Oregon Health & Science University (OHSU) Institutional Review Board. Patients with SpA attending the Uveitis or Rheumatology Clinics at OHSU were recruited to this study and informed consent was obtained before samples were collected. SpA was diagnosed based on the calculation of a likelihood score, as described by Rudwaleit and colleagues [2]. A diagnosis of SpA is made if the likelihood ratio product for all positive factors exceeds 200 [3,4]. Because patients were attending an eye disease clinic, joint disease activity was not formally assessed. However, the likelihood ratio indicates a 90% probability that the subjects have SpA. Ulcerative colitis in one patient was permitted in the SpA group because it is known that SpA may co-exist with inflammatory bowel disease [4]. One patient had psoriasis. All other autoimmune diseases were excluded. Chronic systemic conditions were allowed, as were medications for co-existent morbidities. Systemic immunomodulatory therapy was not permitted. Only one patient is known to have received a TNF inhibitor (etanercept), and this had been discontinued two months prior to the blood draw for this study.

Gene expression in these subjects was compared with that in 25 healthy control subjects without a history of autoimmune disease. Tables 1, 2 and 3 contain demographic and clinical information for the SpA and healthy control subjects. Male subjects in the SpA group outnumbered females as is characteristic of this disease. Neither SpA nor control subjects were on oral corticosteroids or other immunomodulatory therapy. Samples were processed and the results analyzed as two separate datasets, a primary set and validation set, at two different times.

**Table 1:** Dataset characteristics

	Dataset 1		Dataset 2		Combined sets	
			
	SpA	Control	SpA	Control	SpA	Control
**Age (mean ± SD)**	51.8 ± 12.8	48.8 ± 21.4	46.7 ± 14.5	35.4 ± 9.7	49.7 ± 12.9	41.8 ± 17.4
**Years since SpA diagnosis (mean ± SD)**	16.2 ± 14.9		8.2 ± 11.2		13.7 ± 14.0	
**Males/females**	9/2	4/8	4/3	1/12	13/5	5/20

The age and disease duration data have been summarized to protect subject privacy. SD = standard deviation; SpA = axial spondyloarthropathy.

**Table 2:** Individual SpA subject characteristics

Subject	Dataset	Gender	Race	SpA Likelihood ratio	Medications
1	1	M	Asian	4073	None (Etanercept withdrawn for 2 months)
2*	1	M	Caucasian	204	Simvastatin, cyclobenzaprine, aspirin, indomethacin, atenolol, lansoprazole, hydrocodone
3	1	M	Caucasian	204	_
4	1	M	Caucasian	4073	_
5	1	M	Caucasian	934	_
6	1	M	Asian	204	_
7	1	M	Caucasian	4073	Metformin, glipizide, atorvastatin, lisinopril, nifedipine, Lantus insulin, Novo Log, sulfasalazine, indomethacin
8	1	M	Caucasian	558	Ibuprofen
9	1	F	Caucasian	11383	Piroxicam, indomethacin
10	1	F	Caucasian	7115	Rofecoxib
11	1	M	Caucasian	255	Acetaminophen, ramipril, omeprazole, aspirin, atorvastatin
12	2	M	Caucasian	4073	Insulin, nifedipine, glipizide, lisinopril, metformin, Vicodin, Flexeril, indomethacin, sulfasalazine
13	2	F	Caucasian	1039	Celecoxib, trazodone, venlafaxine, ranitidine
14	2	F	Caucasian	204	Tobramycin
15	2	F	Asian	4073	Alendronate
16	2	M	Asian	204	_
17	2	M	Caucasian	20774	Phenylbutazone
18	2	M	Caucasian	2308	Alendronate sodium

*coexistent ulcerative colitis. F = female; M = male; SpA = axial spondyloarthropathy.

**Table 3:** Individual control subject characteristics

Subject	Data-set	Gender	Race	Medications
19	1	F	Caucasian	Alphagan OP, Xalatan OP
20	1	F	Caucasian	_
21	1	F	Caucasian	Atorvastatin, losartan, atenolol, aspirin, hydrochlorothiazide
22	1	M	Caucasian	Atorvastatin, glargine, lisinopril, fluoxetine, amlodipine, Systane OP
23	1	F	Caucasian	Levothyroxine, sertraline
24	1	M	Caucasian	Diazepam, simvastatin, aspirin
25	1	M	Caucasian	Ibuprofen
26	1	F	Caucasian	_
27	1	F	Caucasian	Sertraline, desogestrel/ethinyl estradiol
28	1	M	Caucasian	Ibuprofen, diazepam, acetaminophen/aspirin, esomeprazole, sumatriptan
29	1	F	Caucasian	_
30	1	F	Asian	Trazodone, sertraline, levonorgestrel/ethinyl, estradiol, atorvastatin, ibuprofen, acetaminophen
31	2	F	Caucasian	Acetaminophen, ibuprofen
32	2	F	Caucasian	_
33	2	F	Mixed*	_
34	2	F	Caucasian	Ibuprofen
35	2	F	Caucasian	Etonogestrel/ethinyl estradiol VA
36	2	F	Caucasian	_
37	2	F	Caucasian	Etonogestrel/ethinyl estradiol VA, cetirizine
38	2	F	Caucasian	Desogestrel/ethinyl estradiol
39	2	F	Caucasian	_
40	2	F	Caucasian	Estradiol 0.01% cream, levonorgestrel
41	2	M	Caucasian	_
42	2	F	Caucasian	Duloxetine, valacyclovir, cyclobenzaprine
43	2	F	Caucasian	_

*mixed race is seven out of eight Caucasian and one out of eight African-American.

### Gene expression microarray

Unfractionated whole blood collection and RNA isolation were performed using the PAXgene Blood RNA Isolation System (PreAnalytiX, a Qiagen BD Company, Valencia, CA, USA) according to the manufacturer's recommendation. Microarray assays were performed in the Affymetrix Microarray Core, a unit of the OHSU Gene Microarray Shared Resource. Total RNA was amplified and labeled using a one-cycle target-labeling method modified to reduce globin mRNA targets (GeneChip Globin Reduction Protocol rev.1; Affymetrix, Inc., Santa Clara, CA, USA) and hybridized according to the manufacturer. The high density, human GeneChip^® ^probe arrays (HG-U133 Plus 2.0, Affymetrix, Inc, Santa Clara, CA, USA) were used. Each array contains 54,000 probe sets designed to analyze the expression of 47,000 human transcripts and variants.

Hybridized arrays were processed using the Fluidics Station 450 (Affymetrix, Inc, Santa Clara, CA, USA) and distribution of fluorescence was measured using the Gene Chip Scanner 3000 (Affymetrix, Inc, Santa Clara, CA, USA). Cell fluorescence intensity (CEL) files were generated using the Gene Chip Operating System (GCOS) software version 1.2 (Affymetrix, Inc, Santa Clara, CA, USA).

### Statistical analysis

The 'affy' and 'gcrma' packages of Bioconductor [5] were used to preprocess and normalize the data following import of CEL files into the R statistical package (Affymetrix, Inc, Santa Clara, CA, USA). The GC Robust Multiarray Analysis (GC-RMA) was used to adjust perfect match (PM) probe data for background noise [6]. Normalization was performed on adjusted PM data with an algorithm based on rank invariant probes [7]. After normalization, differential gene expression between groups was assessed by Significance Analysis of Microarrays (SAM) [8]. Differential expression was defined as a 1.5-fold change with a q value less than 5%. The q value is a Bayesian equivalent to the false discovery rate adjusted *P *value [9]. Statistical analysis was performed at an array probe set level; transcript counts were corrected for the presence of multiple probe sets. These data have been used to illustrate an analytical approach described in a statistical methods paper [10] and the controls were also used in a parallel study on gene expression in patients with sarcoidosis [11]. The raw and normalized data have been deposited in the Gene Expression Omnibus repository [GEO: GSE18781] [12].

As males predominated among the SpA subjects and females were more common in the control group, we took additional caution to exclude conclusions attributable to gender. To identify possible gender effects on gene expression levels that might confound interpretation of the intergroup comparisons, an analysis was conducted to determine which of the following four linear models best fit the data for each probe set: (1) a model in which gene expression is impacted by disease state alone; (2) a model in which gender is the sole influence on gene expression; (3) a model in which, after controlling for gender effects, the principal effects are due to disease state; and (4) a model in which the interaction between disease state and gender also influences the results. For this analysis, data from both sets were first renormalized using the quantile normalization method [13]. The well-established Akaike's information criterion [14] was then used to choose the best among four models for each probe set shown in Tables 4 and 5 based on likelihood calculations.

**Table 4:** Putative immune signature in SpA

		Set 1		Set 2		
				
Gene symbol	Gene name	Fold change	Q (%)	Fold change	Q (%)	Function
ALOX12*	Arachidonate 12-lipoxygenase	1.7	2.4	1.9	2.1	Arachidonic acid metabolism; inflammatory response
BCL6*	B-cell CLL/lymphoma 6	1.8	2.4	1.7	1.5	Pleiotropic action in immune response. Inhibits B cell apoptosis
*CLU*	Clusterin	1.5	2.4	1.9	2.1	Complement regulatory action
*CR1*	Complement component (3b/4b) receptor 1	1.5	2.4	1.9	1.0	Complement receptor, regulates B cell apoptosis, immune complex clearance
DEFA4*	Defensin, alpha 4, corticostatin	2.1	2.4	4.2	1.2	Non-specific immune response
*FAM3B*	Family with sequence similarity 3, member B	4.9	2.4	3.6	1.0	IL1-like activity
GRB10*	Growth factor receptor-bound protein 10	1.8	2.4	1.5	1.0	Regulator of nuclear factor kappa B (NFKB)
IL1R1*	Interleukin 1 receptor type I	1.6	2.4	2.0	0.4	Binds to IL1
IL1R2*	Interleukin 1 receptor, type II	1.6	2.4	1.8	1.5	Decoy target for IL1
MAPK14*	Mitogen-activated protein kinase 14	1.5	2.4	1.6	1.8	Part of the MAPK cascade
*NCR3*	Natural cytotoxicity triggering receptor 3	-2.5	4.9	-1.7	1.5	Required for NK cell-mediated induction of dendritic cell maturation
NLRP2/NALP2*	NLR family, pyrin domain containing 2	-2.5	4.3	-1.7	1.5	Part of the inflammasome; inhibits NFkB. Causes caspase-1 activation
*PTGS1/COX1*	Cyclooxygenase 1	1.9	2.4	1.8	2.1	Prostaglandin synthesis.
*SELP*	Selectin P (CD62)	1.7	2.4	1.6	4.1	Extra-lymphoid T cell recruitment. Mediates Endothelial cell and leucocyte interaction
SLPI*	Secretory leukocyte peptidase inhibitor	2.0	2.4	2.4	1.0	Antimicrobial activity; innate host defense mechanism
*SOD2*	Superoxide dismutase 2	1.7	2.4	2.8	1.5	Free radical scavenging enzyme involved in defense against oxidative stress
*SPARC*	Secreted protein, acidic, cysteine-rich (osteonectin)	3.1	2.4	2.3	0.8	Involved in T cell activity and ossification
THBD*	Thrombomodulin	1.5	2.4	1.7	3.1	Innate immune response activity
*THBS1*	Thrombospondin 1	2.0	2.4	2.0	2.5	Glycoprotein
*TREM1*	Triggering receptor expressed on myeloid cells-like 1	1.9	2.4	2.1	2.5	Amplifies response of NLRP2

Significantly differentially expressed genes with a recognized immune or inflammation-related function present in Set 1 and Set 2. Functional annotations were obtained from Online Mendelian Inheritance in Man database [32]. *Secondary analysis indicates that expression level changes are more apparent in males.

**Table 5:** Bone remodeling signature

		Set 1		Set 2		
				
Gene symbol	Gene name	Fold change	Q (%)	Fold change	Q (%)	Function
*BMP6*	Bone morphogenetic protein 6	1.5	2.4	1.7	1.5	Involved in ossification, osteoblast differentiation
CTNNAL1*	Catenin (cadherin-associated protein), alpha-like 1	3.0	2.3	2.3	2.1	Analogous to α-catenin which inhibits β-catenin. Á catenin inhibits wnt/catenin pathway
*KREMEN1*	Kringle containing transmembrane protein 1	2.0	2.4	2.0	2.5	Negative regulator of wnt/catenin pathway
*PCSK6*	Proprotein convertase subtilisin/kexin type 6	3.1	2.4	2.0	1.8	Regulator of BMP6

*Secondary analysis indicates that expression level changes are more apparent in males.

### Pathway analysis of gene expression results

Each gene was studied using a network analysis module within MetaCore™ bioinformatics software (GeneGo Inc, St. Joseph, MI, USA) [15] to identify known functional associations between genes identified in our study and other genes or pathways. These curated networks may include transcription factors, receptors, and enzyme cascades.

## Results

Gene expression microarray analysis was performed on whole blood collected from two independent sets of SpA and control subjects. Our analysis of Set 1 identified 556 probe sets that were upregulated and 962 probe sets that were downregulated in subjects with SpA compared with healthy control subjects. Because some transcript levels were evaluated by multiple probe sets on the microarray chip, the chosen probe sets corresponded to 369 upregulated gene transcripts and 721 downregulated gene transcripts. In Set 2, 704 probe sets (550 gene transcripts) were upregulated; 14 probe sets (7 gene transcripts) were downregulated in patients with SpA relative to the control subjects. Heat maps illustrate differences between the groups [see Additional data file 1]. There were 124 probe sets (92 known and 10 unidentified gene transcripts) that were classified in both sets as upregulated in SpA subjects; 10 probe sets (3 known and 2 unidentified gene transcripts) were downregulated in both sets [see Additional data file 2].

We conducted a literature search using National Center for Biotechnology Information databases, including PubMed [16], on all significantly over- or underexpressed gene transcripts to determine their biological functions. Within the group of transcripts identified in both sets, there were 20 gene transcripts involved in immunity or inflammation that might constitute part of the immune signature in SpA. Table 4 presents these transcripts with functional annotations. In particular, we found upregulation of IL-1 receptors and the downregulation of a potential regulator of the IL-1 pathway, NLRP2. Other upregulated transcripts of interest included 'secreted protein acidic and rich in cysteine' (SPARC) and secretory leukocyte peptidase inhibitor (SLPI). Four gene transcripts that have a role in bone remodeling, including kremen 1, were differentially expressed (Table 5). These might form part of a bone remodeling signature for SpA.

Because of a disproportionate number of females in the control group, we conducted a *post hoc *analysis of variance on the effect of gender. Four models based on different effects of gender and disease state on gene expression were considered. Akaike's information criterion was used to select the model that best fits the data for each probe set. For 14 of the 24 genes included in this secondary analysis, Akaike's information criterion selected the model that assigned the principle expression differences to the disease state after correcting for a gender effect (model 3 in the methods section). The model selected for the remaining 10 genes (marked with an asterisk in Tables 4 and 5) also included an interaction effect of disease state and gender (model 4). For these genes, male subjects with SpA had higher fold-changes than both control subjects and female subjects with SpA, and we cannot exclude a possible effect of gender on the level of transcript expression. However, as an example, even if the downregulation of NLRP2 is a result of the male predominance in the disease group, it would nonetheless represent a novel insight into the male predisposition to SpA.

## Discussion

There are few published studies of gene expression in SpA. Our study reveals a number of genes that are differentially expressed in peripheral blood of patients with SpA and that can be related to the current understanding of its pathogenesis. Our study differs from prior studies in a variety of methodological ways including the number of transcripts studied (more than 47,000 per subject), the exclusion of patients on disease-modifying medications, the use of whole blood, which avoids the potential artifact induced by isolating leukocytes or leukocyte subsets, and pathway analysis *in silico*. Use of a primary dataset and an independent validation dataset provides additional robustness. Utilizing a false discovery rate calculation limits the possibility of false positives due to chance alone.

Almost all of the transcripts identified as having increased or decreased expression [see Additional data file 2] deserve comment with regard to the pathogenesis of SpA, but space precludes such a thorough discussion. We have selected a small number of transcripts for additional comment. The detection of a set of gene transcripts that may have a role in the immune response and are differentially expressed in both datasets suggests the presence of an 'immune signature' in SpA. Prior work has strongly implicated the IL-1 family in the pathogenesis of SpA. Increased IL-1β mRNA has been found in peripheral blood profiling in individuals with spondyloarthropathy [17]. Genetic studies have found that polymorphisms in the IL-1 gene family are associated with ankylosing spondylitis [18] and psoriatic arthritis [19]. The finding that both IL-1 receptor (IL-1R) 1 and IL-1R2 are increased at a transcript level suggests a possible correlation with a genetic association between *ERAP1 *(*ARTS1*) polymorphisms and ankylosing spondylitis [20]; ERAP1 is a proteinase believed to lessen immune responses by cleaving receptors for cytokines including IL-1. Triggering receptor expressed on myeloid cells (TREM)-1 has also previously been implicated in the pathogenesis of ankylosing spondylitis [21]. The detection of transcripts that have independently been implicated in SpA adds to the credibility of gene expression microarray analysis as a technique to identify causal factors in this disease.

SLPI has not previously been implicated in the pathogenesis of SpA. SLPI, however, downregulates the synthesis of TNFα [22] and, as such, may well play an important role in the pathogenesis of this disease that often responds markedly to TNF inhibition. SPARC, which is also known as osteonectin, has been implicated in the pathogenesis of scleroderma [23], but not SpA. SPARC could logically be listed as a contributor to bone remodeling (see below), but it also negatively regulates dendritic cell migration and T cell activation [24].

**Figure 1:**
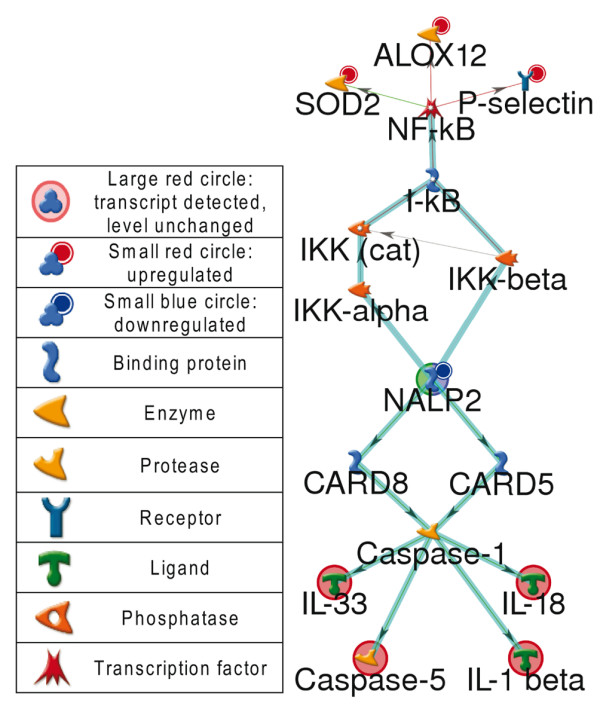
Network illustrating possible role of NALP2 (NLRP2) in SpA via routes leading to NFκB or caspase-1 activation. NLRP2 gene expression is reduced two-fold in axial spondyloarthropathy (SpA) compared with controls. Image generated by GeneGo Metacore™ software [15].

The reduced expression of Nod-Like receptor family, pyrin domain containing 2 (NLRP2 or NALP2) is a novel observation and is especially intriguing. NLRP2 is a component of some inflammasomes [25] and is a member of the NLR family of proteins many of which function as danger-associated molecular pattern receptors of the innate immune system. Polymorphisms in other NLR and related genes have been implicated in diseases that share clinical features with SpA, including Behçet's disease, Crohn's disease, and psoriatic arthritis. Polymorphisms or mutations in genes encoding components or regulators of inflammasomes are associated with several autoinflammatory diseases. NLRP2 functions as an intracellular pattern recognition receptor whose downstream function includes activation of caspase 1 and inhibition of nuclear factor kappa B, both of which lead to regulation of IL-1β (Figure 1) [26,27]. The downregulation of NLRP2 may therefore lead to upregulation of IL-1β, which in turn may regulate IL-1R expression [27]. There is no *a priori *reason to believe that the expression of a gene such as NLRP2 is affected by gender. If NLRP2 is indeed under expressed in males, this downregulation may be an important clue to the male predominance in this disease.

Ossification is the hallmark of SpA, but there is also ongoing bone resorption with up to 56% of patients becoming osteopenic and a significant proportion becoming osteoporotic [28]. The wnt-catenin pathway and its primary regulator Dickkopf 1 (DKK-1) regulate the balance between osteoblast and osteoclast function [29]. The upregulation of kremen1 in our data suggests negative regulation of the wnt-catenin pathway via its interaction with DKK-1. The net effect of this and other factors may be bone resorption [30]. Endogenous bone morphogenetic protein 6 (BMP6) has been described in a mouse model of enthesis ossification and shown to promote osteoblast differentiation. Inhibition of BMP6 prevents the onset and progress of an SpA-type model of arthritis [31].

This study has some limitations. Firstly, although the diagnosis of SpA was made using a validated method, it was not possible to grade disease activity because most patients were attending an eye clinic. Patients did not routinely have X-rays or MRI scans of the pelvis. However, nearly 100% of the patients had inflammatory lower back pain confirmed by a rheumatologist. Secondly, the control group consisted of volunteers with females outnumbering males. There was a predominance of males in the SpA group as is expected in this condition. Gender differences were apparent for a number of differentially expressed genes located on sex chromosomes. These gender-linked genes could be readily identified on the basis of their chromosomal location and they are not known to contribute to inflammation [see Additional data file 2]. A *post hoc *analysis was conducted on the transcripts selected as having higher or lower expression levels in SpA subjects to identify those that were also influenced by gender.

Statistical tests on the effect of gender and/or disease on gene expression revealed that disease, rather than the disproportionate number of males in the group with SpA, accounted for the differences in gene expression. However, gender does play a role in SpA, because the vast majority of patients with SpA are male. For some transcripts the overexpression or underexpression of a particular transcript in SpA is more apparent in males. The directional consistency of differences revealed by the initial SAM analysis and the secondary analysis add further support to our findings.

## Conclusions

Despite the limitations mentioned above, this study has clearly identified a number of novel and intriguing potential contributors to SpA. Gene expression microarray may elucidate pathogenesis, facilitate diagnostic specificity, correlate with pharmacologic responsiveness, and predict prognosis. We based this study in an ophthalmology clinic to test the hypothesis that patients with SpA and active uveitis would express genes in peripheral blood to distinguish those with uveitis from those without uveitis. Our initial evaluation of this hypothesis indicates that a larger database is necessary to determine if such differences exist. This goal will require large databases with careful accrual of clinical data. We believe that the present study represents an important step toward understanding the molecular mechanisms of SpA.

## Abbreviations

BMP: bone morphogenetic protein; CEL: cell fluorescence intensity; DKK-1: Dickkopf-1; GCOS: GeneChip Operating System; GC-RMA: GC Robust Multiarray Analysis; IL-1: interleukin-1; IL-1R: interleukin-1 receptor; NLRP2 (NALP2): Nod-Like Receptor family, pyrin domain containing 2; OHSU: Oregon Health & Science University; PM: perfect match; SAM: Significance Analysis of Microarrays; SLPI: secretory leukocyte peptidase inhibitor; SpA: axial spondyloarthropathy; SPARC: secreted protein acidic and rich in cysteine (also known as osteonectin); TNF: tumor necrosis factor; TREM-1: triggering receptor expressed on myeloid cells 1.

## Competing interests

CH has an equity interest (less than $5,000) in Affymetrix Inc. None of the other authors has any competing interests.

## Authors' contributions

CA recruited subjects and obtained informed consent, drew blood, conducted clinical data entry, and reviewed the manuscript. CH conducted experimental design, supervised microarray assays, conducted data interpretation, and contributed to the manuscript. DC conducted statistical analysis, and contributed to the manuscript. JL recruited subjects and obtained informed consent, drew blood, conducted clinical data entry, and reviewed the manuscript. JR conducted experimental design, examined patients, conducted data interpretation, edited the manuscript, and supervised the entire project. JS conducted experimental design, provided oversight for human subject research, conducted data interpretation, and edited the manuscript. SP conducted experimental design and database design, oversaw RNA extraction, conducted data interpretation, and contributed to manuscript editing. SS examined patients, analyzed data, and drafted the manuscript. TD extracted RNA from blood samples, and reviewed the manuscript. TM contributed to the manuscript.

## Supplementary Material

Additional file 1 PDF file containing heatmaps illustrating expression of genes distinguishing control and axial spondyloarthropathy (SpA) peripheral blood in Set 1 and Set 2. Click here for file

Additional file 2 PDF file containing a table that lists probe sets indicating genes with significantly (q < 5%) higher or lower expression in patients with axial spondyloarthropathy compared with control subjects in Set 1 and Set 2. Click here for file
