# Supplementary material for: Insights in to the pathogenesis of axial spondyloarthropathy based on gene expression profiles
Source: Arthritis Res Ther. 2009 Nov 9;11(6):R168. doi: 10.1186/ar2855 (PMC3003511; doi:10.1186/ar2855)

Supplementary Figure 1: Heatmaps illustrating expression of genes distinguishing control and SpA peripheral blood in Set 1 and Set 2. All genes met the following criteria: >1.5 fold change difference in mean gene expression levels between SpA and control subjects and a significance analysis of microarrays (SAM) q value of < 5%. Each column represents gene expression in one subject and each row represents the signal from one probe set. Yellow indicates genes expressed at higher levels in SpA subjects; blue describes lower levels. The numeric data are provided in Supplementary Table 1.

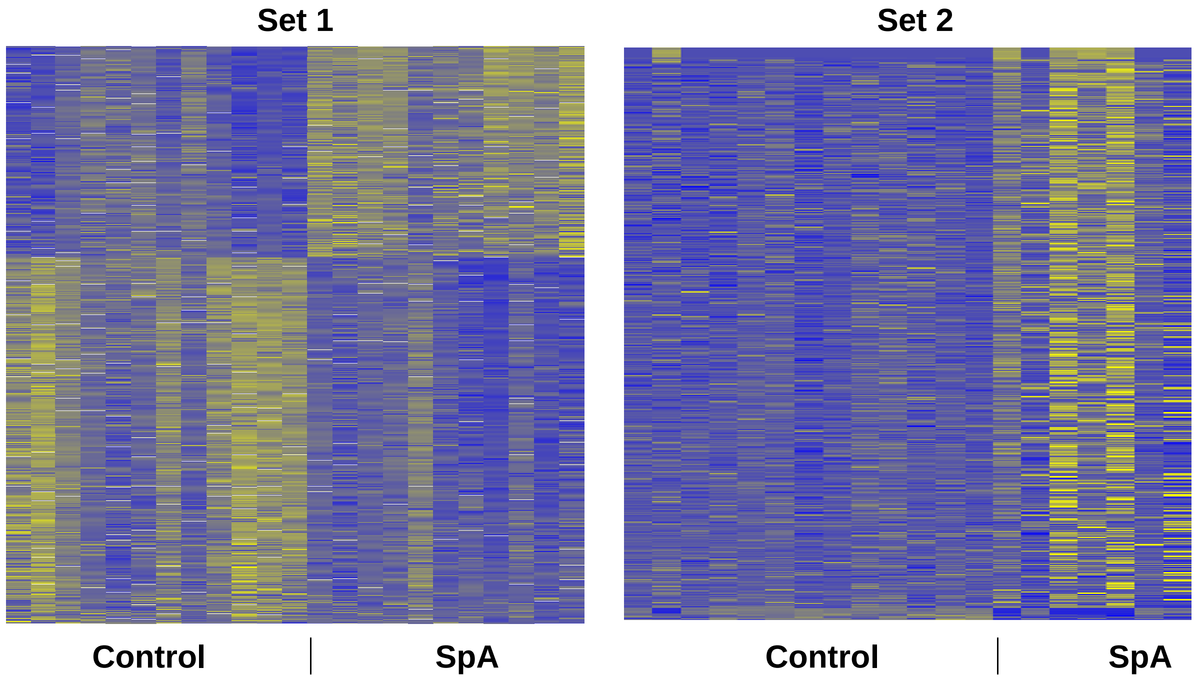

Supplement: Additional file 1 — PDF file containing heatmaps illustrating expression of genes distinguishing control and axial spondyloarthropathy (SpA) peripheral blood in Set 1 and Set 2. [file ar2855-S1.pdf]
