# Supplementary material for: Insights in to the pathogenesis of axial spondyloarthropathy based on gene expression profiles
Source: Arthritis Res Ther. 2009 Nov 9;11(6):R168. doi: 10.1186/ar2855 (PMC3003511; doi:10.1186/ar2855)
Supplement: Additional file 2 — PDF file containing a table that lists probe sets indicating genes with significantly (q < 5%) higher or lower expression in patients with axial spondyloarthropathy compared with control subjects in Set 1 and Set 2. [file ar2855-S2.pdf]

Supplementary Table 1

Probe sets indicating genes with significantly (q <5%) higher or lower expression in patients with axial spondyloarthropathy compared to control subjects in Set 1 and Set 2.

*X- or Y-linked gene.

Probe Set ID	Gene Symbol	Gene Title	Set 1		Set 2	
			Fold Change	q value (%)	Fold Change	q value (%)
201909_at	RPS4Y1*	ribosomal protein S4, Y-linked 1	7.5	1.7	1.6	1.8
205000_at	DDX3Y*	DEAD (Asp-Glu-Ala-Asp) box polypeptide 3, Y-linked	7.5	1.7	1.8	1.8
204409_s_at	EIF1AY*	eukaryotic translation initiation factor 1A, Y-linked	21.6	1.7	3.2	1.2
204410_at	EIF1AY*	eukaryotic translation initiation factor 1A, Y-linked	14.4	1.9	3.2	1.2
206700_s_at	JARID1D*	jumonji, AT rich interactive domain 1D	3.7	1.9	1.7	2.1
244482_at	EIF1AY*	Eukaryotic translation initiation factor 1A, Y-linked	8.1	1.9	3.5	1.0
223646_s_at	CYorf15B*	chromosome Y open reading frame 15B	5.1	2.1	1.5	2.5
228492_at	USP9Y*	ubiquitin specific peptidase 9, Y-linked (fat facets-like, Drosophila)	4.5	2.1	1.6	2.5
206624_at	USP9Y*	ubiquitin specific peptidase 9, Y-linked (fat facets-like, Drosophila)	5.9	2.1	1.5	2.5
214131_at	CYorf15B*	chromosome Y open reading frame 15B	3.2	2.4	1.6	2.5
223645_s_at	CYorf15B*	chromosome Y open reading frame 15B	5.3	2.4	1.6	3.1

205001_s_at	DDX3Y*	DEAD (Asp-Glu-Ala-Asp) box polypeptide 3, Y-linked	7.4	2.4	1.8	2.5
207269_at	DEFA4	defensin, alpha 4, corticostatin	2.1	2.4	4.2	1.2
210322_x_at	UTY*	ubiquitously transcribed tetratricopeptide repeat gene, Y-linked	5.0	2.4	2.2	1.8
220784_s_at	UTS2*	urotensin 2	22.8	2.4	11.0	4.1
220785_at	UTS2*	urotensin 2	11.8	2.4	9.9	4.1
211149_at	UTY*	ubiquitously transcribed tetratricopeptide repeat gene, Y-linked	2.9	2.4	1.8	2.1
202468_s_at	CTNNAL1	catenin (cadherin-associated protein), alpha-like 1	3.7	2.4	2.3	2.1
227194_at	FAM3B	family with sequence similarity 3, member B	4.9	2.4	3.6	1.0
207808_s_at	PROS1	protein S (alpha)	3.7	2.4	1.8	3.6
216956_s_at	ITGA2B	integrin, alpha 2b (platelet glycoprotein IIb of IIb/IIIa complex, antigen CD41)	3.7	2.4	1.7	2.1
205033_s_at	DEFA1 /// DEFA3 /// LOC728358	defensin, alpha 1 /// defensin, alpha 3, neutrophil-specific	1.7	2.4	2.3	2.5
207414_s_at	PCSK6	proprotein convertase subtilisin/kexin type 6	3.1	2.4	2.0	1.8
207601_at	SULT1B1	sulfotransferase family, cytosolic, 1B, member 1	2.3	2.4	2.2	4.1
215150_at	YOD1	YOD1 OTU deubiquinating enzyme 1 homolog (S. cerevisiae)	5.5	2.4	1.8	3.6

219090_at	SLC24A3	solute carrier family 24 (sodium/potassium/calcium exchanger), member 3	4.3	2.4	2.0	1.0
212651_at	RHOBTB1	Rho-related BTB domain containing 1	1.9	2.4	1.8	3.1
204628_s_at	ITGB3	integrin, beta 3 (platelet glycoprotein IIIa, antigen CD61)	2.8	2.4	1.8	1.2
202729_s_at	LTBP1	latent transforming growth factor beta binding protein 1	2.5	2.4	1.5	3.1
203021_at	SLPI	secretory leukocyte peptidase inhibitor	2.0	2.4	2.4	1.0
206698_at	XK*	X-linked Kx blood group (McLeod syndrome)	2.3	2.4	3.2	1.0
206494_s_at	ITGA2B	integrin, alpha 2b (platelet glycoprotein IIb of IIb/IIIa complex, antigen CD41)	2.5	2.4	1.8	1.8
1560800_at	---	MRNA; cDNA DKFZp667C0715 (from clone DKFZp667C0715)	2.1	2.4	1.7	3.6
205110_s_at	FGF13	fibroblast growth factor 13	2.1	2.4	2.0	3.1
214073_at	ALDH1A3	Aldehyde dehydrogenase 1 family, member A3	2.2	2.4	2.4	2.5
1556923_at	ACOX1	Acyl-Coenzyme A oxidase 1	2.0	2.4	1.6	2.1
206493_at	ITGA2B	integrin, alpha 2b (platelet glycoprotein IIb of IIb/IIIa complex, antigen CD41)	2.3	2.4	1.8	2.1
212667_at	SPARC	secreted protein, acidic, cysteine-rich (osteonectin)	3.1	2.4	2.3	0.8
201058_s_at	MYL9	myosin, light chain 9, regulatory	2.4	2.4	2.8	1.8
209369_at	ANXA3	annexin A3	2.0	2.4	2.1	3.1

228950_s_at	GPR177	G protein-coupled receptor 177	1.9	2.4	2.1	1.2
204627_s_at	ITGB3	integrin, beta 3 (platelet glycoprotein IIIa, antigen CD61)	2.6	2.4	2.1	2.1
1552583_s_at	ABCC13	ATP-binding cassette, sub-family C (CFTR/MRP), member 13	1.6	2.4	5.0	3.6
201108_s_at	THBS1	thrombospondin 1	2.0	2.4	2.0	2.5
228949_at	GPR177	G protein-coupled receptor 177	1.7	2.4	1.8	1.8
205003_at	DOCK4	dedicator of cytokinesis 4	1.6	2.4	2.2	1.8
228753_at	---	Transcribed locus, moderately similar to XP_001148836.1 hypothetical protein [Pan troglodytes]	1.9	2.4	1.8	1.5
223670_s_at	HEMGN	hemogen	2.0	2.4	3.4	2.1
213338_at	TMEM158	transmembrane protein 158	2.1	2.4	2.2	2.5
230354_at	---	Transcribed locus	1.5	2.4	1.9	3.6
221958_s_at	GPR177	G protein-coupled receptor 177	1.7	2.4	1.8	1.2
206176_at	BMP6	bone morphogenetic protein 6	1.5	2.4	1.7	1.5
241420_at	LOC728262	Hypothetical protein LOC728262	2.1	2.4	8.9	2.1
236402_at	---	CDNA FLJ42263 fis, clone TKIDN2014570	1.9	2.4	1.6	3.1
229778_at	C12orf39	Chromosome 12 open reading frame 39	2.2	2.4	1.7	1.5
1566964_at	---	---	2.6	2.4	1.7	3.1
1557924_s_at	ALPL	alkaline phosphatase, liver/bone/kidney	2.7	2.4	2.4	1.2

201904_s_at	CTDSPL	CTD (carboxy-terminal domain, RNA polymerase II, polypeptide A) small phosphatase-like	1.6	2.4	1.8	2.1
230972_at	ANKRD9	ankyrin repeat domain 9	2.2	2.4	2.2	2.5
229421_s_at	FLJ20273	RNA-binding protein	2.0	2.4	1.5	2.1
232080_at	HECW2	HECT, C2 and WW domain containing E3 ubiquitin protein ligase 2	2.5	2.4	1.9	1.8
237340_at	SLC26A8	Solute carrier family 26, member 8	2.0	2.4	3.0	0.8
1559756_at	DKFZp667F0711	Hypothetical protein DKFZp667F0711	2.0	2.4	1.9	1.0
207206_s_at	ALOX12	Arachidonate 12-lipoxygenase	1.7	2.4	1.9	2.1
206167_s_at	ARHGAP6	Rho GTPase activating protein 6	1.6	2.4	1.8	2.5
238983_at	NSUN7	NOL1/NOP2/Sun domain family, member 7	2.0	2.4	2.0	1.2
224534_at	KREMEN1	Kringle containing transmembrane protein 1 /// kringle containing transmembrane protein 1	2.0	2.4	2.0	2.5
225142_at	KIAA1718	KIAA1718 protein	1.6	2.4	1.5	1.2
238320_at	TncRNA	Trophoblast-derived noncoding RNA	3.2	2.4	2.2	1.0
200665_s_at	SPARC	Secreted protein, acidic, cysteine-rich (osteonectin) /// secreted protein, acidic, cysteine-rich (osteonectin)	1.7	2.4	1.9	2.1
208116_s_at	MAN1A1	Mannosidase, alpha, class 1A, member 1	2.0	2.4	1.6	2.1
214074_s_at	CTTN	Cortactin	1.7	2.4	1.6	3.1

1558549_s_at	VNN1	Vanin 1	1.6	2.4	1.8	3.6
207815_at	PF4V1	Platelet factor 4 variant 1	1.8	2.4	2.0	3.1
210999_s_at	GRB10	Growth factor receptor-bound protein 10	1.8	2.4	1.5	1.0
212793_at	DAAM2	dishevelled associated activator of morphogenesis 2	7.0	2.4	1.6	3.6
201059_at	CTTN	Cortactin	1.5	2.4	1.7	3.6
201906_s_at	CTDSPL	CTD (carboxy-terminal domain, RNA polymerase II, polypeptide A) small phosphatase-like	1.7	2.4	1.9	1.8
227309_at	YOD1	YOD1 OTU deubiquinating enzyme 1 homolog (S. cerevisiae)	2.2	2.4	1.5	3.1
206049_at	SELP	Selectin P (granule membrane protein 140kDa, antigen CD62)	1.7	2.4	1.6	4.1
221484_at	B4GALT5	UDP-Gal:betaGlcNAc beta 1,4-galactosyltransferase, polypeptide 5	2.6	2.4	2.1	3.1
223669_at	HEMGN	Hemogen	1.7	2.4	1.9	3.6
210594_x_at	MPZL1	Myelin protein zero-like 1	2.0	2.4	1.5	2.5
215078_at	SOD2	Superoxide dismutase 2, mitochondrial	1.7	2.4	2.8	1.5
1565597_at	FLJ20273	RNA-binding protein	1.6	2.4	2.1	1.8
215240_at	ITGB3	Integrin, beta 3 (platelet glycoprotein IIIa, antigen CD61)	1.9	2.4	1.8	2.1
225369_at	ESAM	Endothelial cell adhesion molecule	1.6	2.4	1.5	2.5
204713_s_at	F5	Coagulation factor V (proaccelerin, labile factor)	1.8	2.4	1.8	2.5

205566_at	ABHD2	Abhydrolase domain containing 2	1.5	2.4	1.7	2.1
203305_at	F13A1	Coagulation factor XIII, A1 polypeptide	1.6	2.4	1.5	1.8
1569190_at	SCLT1	Sodium channel and clathrin linker 1	1.6	2.4	1.6	3.6
206655_s_at	GP1BB /// SEPT5	Glycoprotein Ib (platelet), beta polypeptide /// septin 5	1.6	2.4	1.8	3.6
202948_at	IL1R1	Interleukin 1 receptor, type I	1.6	2.4	2.0	0.4
215977_x_at	GK	Glycerol kinase	1.6	2.4	1.8	1.5
205896_at	SLC22A4	Solute carrier family 22 (organic cation transporter), member 4	1.6	2.4	1.8	1.0
216316_x_at	GK /// GK3P	Glycerol kinase /// glycerol kinase 3 pseudogene	1.6	2.4	1.7	1.8
205127_at	PTGS1	Prostaglandin-endoperoxide synthase 1 (prostaglandin G/H synthase and cyclooxygenase)	1.9	2.4	1.8	2.1
225354_s_at	SH3BGRL2	SH3 domain binding glutamic acid-rich protein like 2	1.7	2.4	1.8	2.1
1555659_a_at	TREML1	Triggering receptor expressed on myeloid cells-like 1	1.9	2.4	2.1	2.5
208792_s_at	CLU	Clusterin	1.5	2.4	1.9	2.1
211372_s_at	IL1R2	Interleukin 1 receptor, type II	1.6	2.4	1.8	1.5
230778_at	---	Transcribed locus	1.6	2.4	1.6	0.8
203888_at	THBD	Tthrombomodulin	1.5	2.4	1.7	3.1
240156_at	RFX2	Regulatory factor X, 2 (influences HLA class II expression)	1.8	2.4	1.7	2.5

57588_at	SLC24A3	Solute carrier family 24 (sodium/potassium/calcium exchanger), member 3	1.9	2.4	1.9	1.2
233312_at	ROPN1L / PODN /LOC728124	Ropporin 1-likehypotheticalprotein LOC728124	1.8	2.4	2.0	1.2
243611_at	MICALCL	MICAL C-terminal like	1.8	2.4	1.7	2.1
203661_s_at	TMOD1	Ttropomodulin 1	1.6	2.4	2.4	2.5
223609_at	ROPN1L	Ropporin 1-like	1.5	2.4	2.1	1.8
216925_s_at	TAL1	T-cell acute lymphocytic leukemia 1	1.5	2.4	2.3	3.1
239701_at	ENTPD1	Ectonucleoside triphosphate diphosphohydrolase 1	1.5	2.4	1.7	1.2
226152_at	TTC7B	Tetratricopeptide repeat domain 7B	1.6	2.4	1.6	2.5
206464_at	BMX	BMX non-receptor tyrosine kinase	1.6	2.4	2.9	2.5
230585_at	---	Transcribed locus	1.7	2.4	2.0	1.2
239464_at	---	---	2.0	2.4	1.8	1.8
233700_at	PPP1R12B	Protein phosphatase 1, regulatory (inhibitor) subunit 12B	1.6	2.4	1.5	2.5
237071_at	---	Transcribed locus	1.5	2.4	1.5	3.6
208488_s_at	CR1	Complement component (3b/4b) receptor 1	1.5	2.4	1.9	1.0
215990_s_at	BCL6	B-cell CLL/lymphoma 6 (zinc finger protein 51)	1.8	2.4	3.0	1.0
1558959_at	---	CDNA FLJ37917 fis, clone CTONG1000137	2.2	2.4	1.7	2.1

207201_s_at	SLC22A1	Solute carrier family 22 (organic cation transporter), member 1	1.6	2.4	2.7	2.1
206877_at	MXD1	MAX dimerization protein 1	1.6	2.4	2.8	1.5
211087_x_at	MAPK14	Mitogen-activated protein kinase 14 /// mitogen-activated protein kinase 14	1.5	2.4	1.6	1.8
1554892_a_at	MS4A3	Membrane-spanning 4-domains, subfamily A, member 3	1.7	2.4	1.8	3.1
224588_at	XIST*	X (inactive)-specific transcript	0.5	2.6	0.3	0.4
224589_at	XIST*	X (inactive)-specific transcript	0.1	3.0	0.2	0.4
221728_x_at	XIST*	X (inactive)-specific transcript	0.4	3.0	0.2	0.4
227671_at	XIST*	X (inactive)-specific transcript	0.4	3.0	0.2	0.4
214218_s_at	XIST*	X (inactive)-specific transcript	0.4	3.0	0.2	0.4
224590_at	XIST*	X (inactive)-specific transcript	0.4	3.0	0.2	0.4
221690_s_at	NLRP2	NLR family, pyrin domain containing 2	0.4	4.3	0.6	1.5
239591_at	---	Transcribed locus	0.1	4.9	0.3	0.5
211010_s_at	NCR3	Natural cytotoxicity triggering receptor 3	0.4	4.9	0.6	0.4
216050_at	---	CDNA: FLJ20931 fis, clone ADSE01282	0.3	4.9	0.5	0.4
